# Senior Dance Experience, Cognitive Performance, and Brain Volume in Older Women

**DOI:** 10.1155/2016/9837321

**Published:** 2016-09-25

**Authors:** Claudia Niemann, Ben Godde, Claudia Voelcker-Rehage

**Affiliations:** ^1^Jacobs Center on Lifelong Learning and Institutional Development, Jacobs University, 28759 Bremen, Germany; ^2^Institute of Human Movement Science and Health, Technische Universität Chemnitz, 09126 Chemnitz, Germany; ^3^Department of Psychology & Methods, Jacobs University, 28759 Bremen, Germany; ^4^Center for Cognitive Science, Bremen University, 28359 Bremen, Germany

## Abstract

Physical activity is positively related to cognitive functioning and brain volume in older adults. Interestingly, different types of physical activity vary in their effects on cognition and on the brain. For example, dancing has become an interesting topic in aging research, as it is a popular leisure activity among older adults, involving cardiovascular and motor fitness dimensions that can be positively related to cognition. However, studies on brain structure are missing. In this study, we tested the association of long-term senior dance experience with cognitive performance and gray matter brain volume in older women aged 65 to 82 years. We compared nonprofessional senior dancers (*n* = 28) with nonsedentary control group participants without any dancing experience (*n* = 29), who were similar in age, education, IQ score, lifestyle and health factors, and fitness level. Differences neither in the four tested cognitive domains (executive control, perceptual speed, episodic memory, and long-term memory) nor in brain volume (VBM whole-brain analysis, region-of-interest analysis of the hippocampus) were observed. Results indicate that moderate dancing activity (1-2 times per week, on average) has no additional effects on gray matter volume and cognitive functioning when a certain lifestyle or physical activity and fitness level are reached.

## 1. Introduction

Over the past few decades, research has shown that physical activity benefits cognitive performance in older adults (e.g., review article by Bherer et al. [1]), affirming that tasks measuring executive functions and controlled processes profit the most (see meta-analysis by Colcombe and Kramer [2]). Furthermore, it has been demonstrated that physical activity also diminishes the risk of suffering from dementia [3] and benefits hippocampal volume and memory performance [4–6].

The majority of these studies focus on cardiovascular activities, such as walking or bicycling. However, motor fitness and coordination training have also been shown to benefit cognitive function [7, 8] and brain volume [9] in older adults (see review articles by Erickson et al. [10] and Voelcker-Rehage and Niemann [11]). Motor fitness represents the skill-related components of physical fitness, which are based on information processing [11]. It refers to the use of the senses (e.g., sight and hearing) for coordinated abilities, together with body parts for performing motor tasks smoothly and accurately [12]. Examples of this include reaction speed, hand-eye coordination, balance, and agility. Training that aims to improve motor fitness is called coordination training. Using dancing as a multimodal type of physical activity that addresses cardiovascular, coordinative, and cognitive demands while providing an attractive leisure activity for older adults may be an effective means to improve cognitive and brain function in this population. We hypothesized that a combination of cardiovascular and coordinative demands would add to the common effects of cardiovascular activity on cognitive performance and brain volume and maximize its impact on neuroplasticity and cognition (for a study on additive effects of cardiovascular activity and environmental enrichment in animals, see Fabel et al. [13]). However, to date, only a small number of studies have investigated the effects of regular dancing activity on cognitive performance with mixed results. None of these studies included neurophysiological measures.

A first prospective study by Verghese and colleagues [14] showed leisure dancing to be associated with a reduced risk of developing dementia. A similar cross-sectional study conducted a few years later, however, did not confirm this result. Adults aged 80 years (standard deviation [SD] = 6.5 years) who engaged in multiple years of nonprofessional social dancing activity did not reveal better cognitive performance in the domains of memory (Free and Cued Selective Reminding Test), verbal fluency, and executive control (Digit Span Task, Digit Symbol Substitution Test, Block Design Test, and Trail Making Test), in comparison to nondancers [15]. Similarly, a pilot study of 13 healthy older women (mean (M) age = 68 years; SD = 8.6 years) did not demonstrate improvements in cognitive performance measured using the Mini-Mental State Examination (MMSE) after a 12-week jazz dance intervention [16]. Recent studies show more positive results. Older adults (M age = 71.69; SD = 1.15 years) with long-term dancing experience exhibited better cognitive performance in fluid intelligence (Raven Standard Progressive Matrices) and attention (Geriatric Concentration Test), in comparison to age-matched inactive controls [17]. Furthermore, the same research group observed an increase in performance in an overall index of cognition (comprised of concentration, attention, and nonverbal learning) in older adults participating in a six-month dancing intervention [18].

To our knowledge, no past research has investigated the relationship between dancing and brain volume in older adults. Additionally, previous studies in this field often compared samples that were dissimilar in measures of lifestyle (e.g., social participation), physical activity behaviour, and fitness, which may have influenced results. Comparing the cognitive effects of senior dancers against a nonsedentary control group allows for the assessment of the impact of dancing experience against a “normative” (physically) active lifestyle. As cognitive decline during late adulthood occurs in parallel with brain volume shrinkage [19, 20], interventions that could diminish age-related brain volume loss are of specific interest. Thus, we were keen to explore whether older women with long-term dancing experience in a senior dancing class showed better cognitive performance and gray matter (GM) volume, in comparison to nonsedentary age-matched controls. Based on previous research performed separately on cardiovascular and coordinated activity and in animals, we expected better performance in the dancing group as compared to the control group, in cognitive aspects that are particularly affected by increasing age, including episodic memory, long-term memory, executive control, and perceptual speed as basic components of fluid intelligence [21]. At the brain level, we assumed a positive effect of long-term dancing experience on GM volume, especially in the hippocampus [6].

## 2. Materials and Methods

### 2.1. Participants

We recruited 57 healthy older women (M age = 72.92 years; SD = 4.50; range = 65–81 years) in Bremen, Germany, through the member registry of the German Federal Association of Senior Dance (Bundesverband Seniorentanz e.V.) and newspaper advertisements. We restricted data collection to women only, due to the failure to recruit enough age-matched male senior dancers in Bremen and to homogenize the sample. Participants were screened for any history of cardiovascular disease, neurological disorders (e.g., self-report of neurological diseases, such as brain tumor, Parkinson's disease, or stroke), other motor or cognitive restriction (a score of less than 27 on the MMSE [22]), or metal implants. All subjects participated voluntarily in the study and provided written, informed consent for the procedures. The study was approved by the Ethics Committee of the German Psychological Society and conformed to the ethical principles for medical research involving human subjects outlined by the World Medical Association Declaration of Helsinki.

To avoid or control for the confounders present in many previous studies, participants were given questionnaires to determine their demographics, handedness, health, habitual physical activity level, and social participation. General intelligence (IQ score) was measured with a test battery from the Berlin Aging Study [21], which included five tests representing five primary intellectual abilities: (a) perceptual speed (Identical Pictures Test), (b) reasoning (test of Figural Analogies), (c) memory (Paired-Associate Learning Test), (d) verbal fluency (Naming Animals), and (e) verbal knowledge (Vocabulary Test). Performance scores were transformed into* T* scores (M = 50, SD = 10) and a mean intelligence index (IQ score) was calculated to correct for differences in general intelligence during the analyses (cf. Colcombe et al. [23]). Habitual physical activity was measured using the German version of the Baecke et al. [24] questionnaire [25] and was expressed in kilocalories/kilo body weight expended per week (kcal/kg*∗*wk) by leisure time and physical activities, according to the recommendations from Ainsworth et al. ([26, 27] range between 10.14 and 30.76 kcal). Energy expenditure in relation to dancing activity was excluded (see below). Social participation was measured according to the questionnaire of participation in 17 everyday activities by Aartsen and colleagues [28] and was expressed as the sum of self-reported social activities over the previous months (range between 44 and 76 activities).

Half of the sample (*n* = 28) included current members of a senior dancing class of the German Federal Association of Senior Dance that were active for at least five years (minimum dancing activity of one time per week). Mean senior dancing experience was 13.38 years (range = 5–34 years; SD = 7.73 years). Senior female dancers participated in dancing classes a mean of 1.59 times per week (range = 1–4 times/week; SD = 0.89) for at least 60 minutes, resulting in approximately 6.70 kcal/kg*∗*wk spent on dancing activity. The other half of the sample (*n* = 29) did not have any dancing experience and served as a nonsedentary control group. Both groups were statistically similar to one another in terms of measures of age, years of formal education, IQ score, BMI, hypertension, estrogen replacement therapy, subjective health, living condition, social participation, physical activity (dancing activity not included), and cardiovascular and motor fitness levels (see Table 1 for M and SD, all variables *p* > .09). Both groups were engaged in similar physical activities, aside from dancing (cf. above). Main activities across the whole sample were bicycling, walking, doing gymnastics or water gymnastics, and swimming.

### 2.2. Fitness Assessments

*Cardiovascular fitness* was assessed by spiroergometry (ZAN 600 from nSpire Health based in Oberthulba, Germany), which is a measurement system of oxygen consumption and indirect calorimetric assessment. Participants were required to obtain consent from their family physician before commencing cardiovascular fitness testing. During spiroergometry, participants completed a submaximal graded exercise test (three minutes of warming up at 10 watts, followed by an increase of 10 watts/min during the test, and a five-minute cooldown) on a bicycle ergometer (Ergoline Ergoselect 100P rpm-independent cycle ergometer manufactured in Bitz, Germany). A medical doctor, in cooperation with a certified sports scientist, secured a ten-lead electrocardiography (ECG) fully digital stress system (Kiss, GE Healthcare, Munich, Germany) to continuously monitor respiration, heart rate, blood pressure, and electrocardiography during the testing protocol. The mean value of oxygen consumption (VO_2_) at the highest complete performance level achieved by the participants, expressed as VO_2_ peak mL/kg, was used for data analysis. The test was stopped after either participants maintained a respiratory exchange ratio of 1 for at least 30 s, due to volitional exhaustion or risk factors occurred. Protocols that were terminated because of risk factors (e.g., blood pressure above 230/115 mmHg, cardiac arrhythmia) or due to volitional fatigue that occurred before the respiratory quotient reached 1 were not used for analysis.

*Motor fitness* was assessed according to the study of Voelcker-Rehage et al. [7], by using a heterogeneous battery of eight motor tests representing three different domains of motor fitness:* movement and reaction speed*,* balance*, and* fine motor coordination*.* Movement speed* was measured using three tests: Foot Tapping Test, Agility Test, and 30  s Chair Stand Test. During the Foot Tapping Test [29], participants sat on a chair and tapped both feet simultaneously across a marker on the floor in front of them (the number of taps within 20 s was counted, with the best of the two trials selected for analysis). The Foot-Up-and-Go Test by Adrian [30] was used to assess agility. Participants sat on a chair, stood up, walked around a cone eight feet in front of them, and then sat back down on the chair (the best out of three trials was selected for analysis). During the 30  s Chair Stand Test [31], participants began by sitting on a chair and continuously got up and sat down (the number of get-ups within 30 s was counted and used for analysis).* Reaction speed* was assessed by the Stick-Fall Test [32], in which participants gripped a falling stick as soon as possible with their dominant and nondominant hand (three trials with right and left hand; falling distance in cm was measured, and the mean of the best trial of the right and left hand was used for data analysis).* Balance* was measured by three tests: the Backwards Beam Walk Test and the One-Leg Stand with eyes open and closed. In the Backwards Beam Walk Test [33], participants walked backwards on three balance beams of differing widths: 6 cm, 4.5 cm, and 3 cm (the number of steps on each beam was counted with a maximum of eight steps per beam). Three trials per beam were performed and the steps of all nine trials were added together for a total score). In the One-Leg Stand [34], participants looked straight ahead while standing on one leg with the other slightly flexed. Compensatory movements of the arms and the lifted leg, but not of the standing leg, were accepted. Participants performed three trials standing on the right and left leg each at a time (duration of standing in seconds with a maximum of 20 s was noted for each trial, and the mean of the best trial with the right and left legs was used for data analysis). The One-Leg Stand with eyes closed was performed accordingly.* Fine motor coordination* was measured by the Purdue Pegboard Test [35]. During this test, participants placed as many pegs as possible into the holes of a pegboard in 30 s. Participants performed this task three times with each hand separately and then with both hands simultaneously (the mean value from the best of the three trials per condition was used for data analysis).

An overall index of motor fitness (mean of the* z*-transformed individual performances within the three domains) was calculated from the individual performances in the three fitness dimensions: speed (mean of the four tests), balance (mean of the three tests), and fine motor coordination.

### 2.3. Cognitive Assessments

*Memory *was measured by the German version of the Auditory Verbal Learning and Memory Test (AVLMT, [36]). For assessing* episodic memory* performance, participants were asked to remember and recall as many words as possible after hearing a list of 15 words. The same list was presented in five consecutive trials for approximately 10 to 15 minutes; the total number of words recalled over the five trials was used for data analysis with a maximum value of 75.* Long-term memory* performance was assessed using a delayed recall trial after approximately 20 to 30 minutes (participants underwent further cognitive testing during this time) without additional presentation of the list (the difference between the number of recalled words for the fifth trial and the number of recalled words for the delayed trial was used for data analysis; the lower the difference, the better the performance; see also [36]).

*Executive control* was measured by a modified version of the Flanker Task with three response conditions, as reported elsewhere [37]. Participants were asked to identify a colored target in the center (red or green) surrounded by four distractors (congruent condition: same color; incongruent condition: competing color; neutral condition: different color (blue)) by pressing a button with either the left index finger (digit N on a German keyboard for red) or right index finger (digit X on a German keyboard for green). Participants performed three blocks of 50 Flanker items, each presented in random order. Further task parameters were a fixation cross-exposure time of 300 ms, an intermediate blank period of 200 ms, a stimulus duration of 500 ms, a reaction period until a response was made or 1000 ms has passed, a mean random trial variance of 150 ms, and an interblock break duration of 10 s. Participants performed 20 practice trials prior to testing to ensure the task was understood.

Performance of the Flanker Task was measured by speed (i.e., reaction time of correct incongruent trials) and accuracy (i.e., percentage of correct incongruent trials). To offset speed against accuracy, a standardized performance index (*q*-score) was calculated based on reaction time and response accuracy measures in the incongruent condition [38]:* q*-score is equal to IQ standardized mean reaction time (RT correct responses/IQ standardized percentage of correct responses) with IQ standardized scores having a mean of 100 and a standard deviation of 15. A lower* q*-score represents faster, more accurate performance on the test.

*Perceptual speed* was measured by a Visual Search Task that used filled and unfilled squares and circles as stimuli [39]. We used the conjunction search condition, where a filled circle (target) had to be found among 2, 8, or 14 unfilled circles and filled squares (both distractors) appearing on the black screen of a computer monitor. Each participant performed four blocks of 50 trials each in a randomized order, with possible combinations of three display sizes (2, 8, or 14 items), target present or absent, and eight target locations (four inner and four outer quadrants, which mattered in target-present conditions only). Further task parameters were a fixation cross-exposure time of 300 ms, an intermediate blank period of 200 ms, a stimulus duration until a response was made or 5000 ms had passed, a mean random trial variance of 150 ms, and an interblock break duration of 30 s. Presence of the target was indicated when participants pressed the digit N on a German keyboard with the right index finger as quickly and as accurately as possible; absence of the target was indicated when participants pressed the digit X on the keyboard with the left index finger. Participants performed 20 practice trials (14 items present) prior to testing to ensure that they understood the task.

Performance of a Visual Search Task was measured by speed (i.e., the reaction time of correct 14-item trials) and accuracy (i.e., the percentage of correct answers for 14-item trials). As above, standardized performance indices (*q*-score) for the 14-item condition were calculated from reaction time (only correct responses) and response accuracy [38].

These cognitive domains were chosen for the current analyses for two reasons: the meta-analysis by Colcombe and Kramer [2] showed that executive control and controlled processes (measured using a Visual Search Task) benefit the most from physical activity and hippocampal volume and memory performance are of particular interest with regard to effects of physical activity in older adults (e.g., [4, 40–42]).

### 2.4. Study Design

Each participant completed the motor and cognitive tests within two laboratory sessions of approximately 1.5 h each. All tasks were administered in a fixed order. On a separate day, structural T1-weighted anatomical brain scans were collected using a 3-Tesla MRI scanner (MPRAGE sequence, TR of 1900 ms, 192 slices with 1 × 1 × 1 mm^3^ isotropic resolution). This was part of a larger MRI protocol, which lasted a total of about 45 minutes.

### 2.5. MRI Data Processing and Analysis

#### 2.5.1. Whole-Brain Voxel-Based Morphometry

We used SPM8 (Statistical Parametric Mapping Version 8; Wellcome Trust Centre for Neuroimaging, University College London, London, UK) running under MATLAB version 7.12 (MathWorks, Sherborn, MA, USA) with help from the VBM8 toolbox (Structural Brain Mapping Group, University of Jena, Jena, Germany; http://dbm.neuro.uni-jena.de/vbm/download/) for preprocessing of the T1-weighted images and determination of voxel-based volume differences between senior dancers and control group participants across the whole brain. We applied standard VBM8 routines and default parameters. The “new segmentation” preprocessing procedure implemented in VBM8 consists of an iterative combination of corrections for bias-field inhomogeneity, high dimensional spatial DARTEL (Diffeomorphic Anatomical Registration Through Exponentiated Lie Algebra) normalization into MNI (Montreal Neurological Institute) space, tissue classification into GM, white matter (WM), and cerebrospinal fluid (CSF). In addition, a modulation step multiplies GM images by the local value derived from the deformation field, thereby preserving within-voxel volumes that may have been altered during the normalization step (VBM8 toolbox manual: http://dbm.neuro.uni-jena.de/vbm/download/). The modulated GM volumes were then smoothed with an isotropic Gaussian kernel of 8 mm full width at half maximum (FWHM) to accommodate inexact spatial normalization. The normalized, modulated, and smoothed GM images were used in the statistical analysis.

Previous research has shown that region-of-interest- (ROI-) based analysis is more sensitive in finding volume differences in the medial-temporal lobe region between study groups [10, 43]. Thus,* hippocampal volume determination* was separately performed with the help of NeuroQuant® software (CorTechs Labs Inc., San Diego, CA; http://www.cortechslabs.com/). The T1-weighted image of each participant was uploaded to the NeuroQuant server, where the brain imaging data were processed and analyzed. NeuroQuant uses a computer-automated analysis involving several preprocessing steps (e.g., skull stripping; inflating the brain to a spherical shape; mapping the spherical brain to a common spherical space shared with the Talairach Atlas brain; identification of brain regions; and deflation of the patient's brain back to its original shape while retaining the identifying information for brain segments). The advantage of this analysis is that it measures volumes of brain structures for several (mainly subcortical) regions. Mapping outputs were visually inspected for segmentation irregularities; no scans had to be excluded from analysis. We used the absolute hippocampal volumes determined by NeuroQuant (left and right hemispheres, separately) and adjusted the raw data for intracranial volume (ICV) to account for head size differences as recommended by Raz and colleagues [44]. We used manual morphometry volume determination for ICV, the same adjustment procedure as within our previous studies [6].

### 2.6. Statistical Analyses

Although both groups were statistically similar in demographic and health variables, we included age (due to the wide age range in this sample) and IQ score (as general intelligence has been proven to impact brain volume, cf. Ritchie et al. [45] and Basten et al. [46]) as covariates in all subsequent analyses to correct for brain volume differences relevant to age and general intelligence.

#### 2.6.1. Cognitive Tasks and Hippocampal Volume

Multivariate analyses of covariance (MANCOVA) were used to test for differences between senior dancers and control group participants in regard to either cognitive performance (Analysis 1: group as an independent variable; the four cognitive tasks of episodic memory, long-term memory, executive control, and perceptual speed as dependent variables) or hippocampal volume (Analysis 2: group as an independent variable; two hippocampal volumes of right and left hemispheres as dependent variables). Effect sizes were calculated by partial eta-square (p*η* ^2^), expressing the amount of variance explained in the dependent variables by the respective effect. For all analyses, the significance level was set to *α* = .05.

#### 2.6.2. Voxel-Based Morphometry Data

A group comparison of GM volumes was performed using both voxel- and cluster-level inference within the framework of the general linear model (GLM), with an absolute threshold of 0.2 to avoid possible partial volume effects near the border between GM and WM. As outlined above, age and general intelligence (IQ score) were included in the model as covariates. One-sided two-sample* t*-tests were performed voxelwise to test for local volume differences between senior dancers and control group participants across the whole brain separately for both directions (senior dancers > control groups; senior dancers < control group). We reported effects for clusters of voxels exceeding a statistical threshold at a voxel level of *p* < .001 (uncorrected) and additionally reported the familywise error (FWE) correction value of the cluster level (correction for multiple comparisons).

#### 2.6.3. Association between Brain Volume and Cognitive Performance

To examine whether brain volume was associated with cognitive performance, we performed a 2-tailed Pearson product-moment correlation analysis to test whether the ICV-adjusted left and right hippocampal volumes correlated with individual performances in the cognitive tasks. The correlation analysis was, again, controlled for age and IQ score. For significant correlations, followup linear regression analyses were used to determine the percentage of explained variance on cognitive performance within the respective brain region.

## 3. Results

### 3.1. Cognitive Tasks

The MANCOVA did not reveal any significant benefit for female senior dancers in the four cognitive tasks covering perceptual speed, executive control, episodic memory, and long-term memory in comparison to nonsedentary age-matched control participants (see Table 2 for M, SD,* F*-, and* p* values of the respective MANCOVAs).

### 3.2. Hippocampal Volume

The MANCOVA with the ICV-adjusted hippocampal volume of the left and right hemispheres revealed no differences between senior dancers and nonsedentary control group participants (see Table 3 for M, SD,* F*-, and* p* values of the respective MANCOVAs).

### 3.3. Voxel-Based Morphometry Data

**Figure 1:**
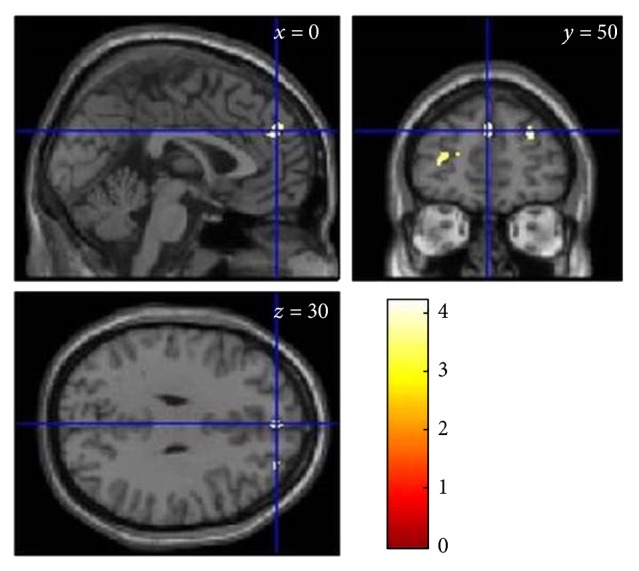
Regions where gray matter volume was larger (nonsignificant on cluster level) in senior dancers than control group participants (see also Table 4).

T-contrasts revealed no clusters in which senior dancers showed larger GM volume than controls when results were controlled for familywise errors. However, the uncorrected analysis clusters in frontal brain regions tended to be larger in senior dancers than in controls (see Figure 1, Table 4). When we tested the opposite contrast—whether brain volume was larger in control group participants than senior dancers—no cluster was found (data not shown).

### 3.4. Additional Correlation Analysis

Across both groups, Pearson product-moment correlation analysis revealed that ICV-adjusted volumes of the left and right hippocampi were negatively correlated with long-term memory performance, indicating a positive relationship between brain volume and cognitive performance (see Table 5). Followup linear regression analyses revealed that individual volume of the hippocampus (both hemispheres together) explained 15.1% of variance in long-term memory performance (left hippocampus:* B* = −0.07; SE* B* = 0.90; *β* = −.12; *p* = .45; right hippocampus:* B* = −1.49; SE* B* = 0.75; *β* = −.31; *p* = .05).

## 4. Discussion

In this study, we investigated whether long-term senior dancing experience in older women aged 65 to 82 years was associated with better cognitive performance in the domains of episodic memory, long-term memory, executive control, and perceptual speed and whether larger GM volume might explain these expected cognitive benefits. In contrast to our expectations, we did not find female senior dancers to show better cognitive performances than nonsedentary controls. Similarly, in the VBM analysis, no brain regions revealed larger volume in senior dancers in comparison to the control group of similar age, education, IQ score, lifestyle and health factors, and fitness levels. Again, ROI-based analysis of hippocampal volume did not show any differences between both groups. Across the whole sample, hippocampal volume explained 15.1% of variance in long-term memory performance.

As outlined in the introduction, findings on dancing activity and cognitive functioning in older adults are mixed. Our results align with a cross-sectional study by Verghese [15], who did not find better performance in memory (Free and Cued Selective Reminding Test) and several tests measuring executive control for experienced dancers in comparison to nondancers. We were also not able to demonstrate any cognitive benefits of multiple years of nonprofessional dancing activity in these domains, most notably for episodic memory and perceptual speed (Table 2). This result is in contrast to Kattenstroth and colleagues [17], who revealed better attentional performance (Nonverbal Geriatric Concentration Test [AKT]), as well as higher scores in a task measuring fluid intelligence (Raven Standard Progressive Matrices) in older adults with long-term dancing experience. As we (as well as [15]) did not measure attention and used other tasks to assess domains of fluid intelligence, one possible explanation might be that cognitive benefits from long-term dancing activity in late adulthood are highly task-specific.

A further explanation for the dissimilar findings in relation to past studies might be based on the activity level of the control group. For example, in a study by Kattenstroth et al. [17], the control group was an inactive sample. They reported no regular sporting activities and revealed lower scores in everyday competence, as well as significantly lower performance in the domains of posture, balance, and motor performance than the older dancers group. In contrast, our control group participants were similarly active to dancers with respect to engaging in physical activities like walking, bicycling, or doing gymnastics and also revealed comparable scores in social participation (Table 1). Likely more important is the observation that, despite the senior dancers group having a higher weekly energy expenditure based on physical activity (regular physical activity plus dancing activity), both groups did not differ in cardiovascular and motor fitness (Table 1). This indicates that the additional dancing activity was not directly associated with improved cardiovascular or motor fitness levels (neither speed nor balance nor fine motor coordination). Cardiovascular fitness levels in both senior dancers and control group participants were age-appropriate. According to norm levels defined by Shvartz and Reibold [47], both groups demonstrated cardiovascular fitness levels slightly above the age-related mean value of VO_2_ peak (17.5 mL/kg). One could postulate that a mean frequency of dancing activity of 1.59 times per week is not enough to induce additional motor and cognitive benefits in senior dancers. Similarly, in the study by Verghese [15], dancers and control group participants differed only in terms of dancing activity, but not in other leisure activity. However, the older dancers in that study revealed better balance performance and gait parameters (but not strength) than the control group [15] and thus exhibited better motor fitness performance.

Thus, it might be reasonable to assume that dancing activity (additionally) benefits cognitive performance only when it is associated with higher cardiovascular and/or motor fitness. Due to the fact that the groups in our sample were similar in fitness levels, it would seem that long-term nonprofessional dancing experience alone does not lead to benefits in fitness and cognitive performance. An overall inactive lifestyle accompanied by lower levels of motor competencies might account for the observed differences between older dancers and controls in the study by Kattenstroth and colleagues [17], thereby offering an explanation for the conflicting findings of association between long-term dancing activity and cognitive performance among the three cross-sectional studies.

Previous research confirmed a positive association between higher levels in cardiovascular fitness and better cognitive performance (e.g., review article by Bherer et al. [1]) and a growing number of studies prove a similar association for motor-related fitness dimensions [7–9, 17, 48, 49]. Our findings may indicate that the specific type or amount of physical activity (like here, e.g., dancing activity) does not seem to determine cognitive benefits in older ages, but rather fitness levels (e.g., thresholds).

In relation to GM volume, larger brain volume in senior dancers as compared to controls was only revealed in the uncorrected analysis (see Table 4 and Figure 1), with this finding restricted to the frontal lobe, which has been found to be particularly associated with physical activity (see review articles by Erickson et al. [10] and Voelcker-Rehage and Niemann [11]). For example, Weinstein and colleagues [50] reported volumes of frontal brain regions to be related to cardiovascular fitness (e.g., left middle frontal gyrus, right inferior frontal gyrus, and precentral gyrus) in a sample of older adults. In addition, they revealed that the volume of the left middle frontal gyrus together with the volume of the precentral gyrus accounted for 17.5% of the variance in spatial working memory performance.

Although physical activity has been shown to be positively related to hippocampal volume or perfusion (for cardiovascular training, see [4, 5]; for coordination training, see [6]), we could not reveal in this study larger hippocampal volume for the investigated group of older female senior dancers in comparison to age-matched controls without dancing experience (Table 3). The absence of differences in hippocampal volume between both groups (similar to no differences in cognitive functioning) might be explained by the similar level of cardiovascular and motor fitness in both groups. Across the whole sample, a larger volume of the hippocampus (especially of the right hemisphere) was related to long-term memory performance (Table 5; see also review article by Yonelinas [51], on the importance of the hippocampus on long-term memory formation). Studies on the association between (cardiovascular) fitness and hippocampal volume, however, still present mixed results.

### 4.1. Limitations

We have to acknowledge certain limitations of this study. First of all, the cross-sectional design is associated with selection biases and cannot support causality. To date, no existing study has used a randomized controlled design to explore differences in brain volume and cognitive performance based on senior dance activity in older adults. Therefore, additional research using randomized controlled trials to test the efficacy of dancing on functional and structural MRI measures in activity-matched older adults is warranted. Secondly, sample size in the current study is moderate (*N* = 57), but comparable to other investigations studying the impact of physical fitness on MRI parameters in older adults (cf. Bugg and Head [52], Colcombe et al. [2]; Colcombe et al. [23]). Thus, we assume that the reported null results are not due to a lack of statistical power. Moreover, cardiovascular and motor fitness parameters did not differ between long-term senior dancers and control group participants. The physical activity level of both groups differed only in that senior dancers participated in a senior dancing class an additional 1.59 times per week, on average. Knowing that fitness level has been shown to positively impact functional and structural MRI parameters might be a precondition for demonstrating differences in physical activity effects in an older adult's brain.

## 5. Conclusions

In this study, we investigated the association of long-term senior dance experience in older women on cognitive functioning and brain volume, in comparison to control group participants who lack dancing experience but are similarly active in other leisure activities and reveal comparable fitness levels. Findings showed that senior dancers did not benefit in the cognitive domains of executive control, perceptual speed, episodic memory, and long-term memory performance. Moreover, dancing experience showed no effects on GM volume. These results might be explained by the comparable fitness levels (cardiovascular fitness and motor fitness) of both study groups. We assume that differences in fitness levels or minimum (threshold) fitness levels have more impact on cognitive functioning and brain volume than the type of performed physical activity.

## Figures and Tables

**Table 1:** Demographic and health information (M and SD) for senior dancers (*n* = 28) and control group (*n* = 29) participants.

Characteristic	Senior dancers		Control group		*p*
	M	SD	M	SD	
Age	73.10	4.32	72.73	4.13	.74
Education	12.70	2.36	13.09	2.96	.59
IQ score	50.54	4.72	51.61	4.90	.40
BMI	26.29	2.76	25.01	3.54	.13
Hypertension	0.46	0.51	0.38	0.49	.53
ERT	0.29	0.46	0.41	0.50	.32
Subjective health	3.72	0.64	3.79	0.73	.71
Social participation	60.82	6.95	59.02	7.64	.36
Living condition	0.36	0.49	0.59	0.50	.09
Physical activity	18.93	7.34	17.89	7.85	.61
Cardiovascular fitness	19.28	3.74	20.18	4.48	.41
Motor fitness	0.08	0.67	−0.08	0.61	.44

Note: age (average age in years), education (years of formal education), IQ score, BMI (body mass index), hypertension (proportion of participants who were diagnosed with hypertensive disorders), ERT (estrogen replacement therapy), proportion of participants who recieved estrogen replacement therapy, social participation, living condition (proportion of participants who reported living with others), physical activity (other than dancing activity; in kcal/kg*∗*wk), cardiovascular fitness (VO_2_ peak mL/kg), and motor fitness (overall index of *z*-scores).

**Table 2:** M and SD for performance in the four cognitive tasks separated for senior dancers (*n* = 28) and controls (*n* = 29), as well as *F*- and *p* values and effect sizes of the respective MANCOVA with age and IQ score as covariates.

Cognitive task	Group	M	SD	*F*(1,55)	*p*	p*η* ^2^
Flanker Task (*q*-score)	Senior dancers	1.01	0.26	0.62	.44	.01
	Control group	1.05	0.28			
Visual Search Task (*q*-score)	Senior dancers	1.06	0.39	0.64	.43	.01
	Control group	1.00	0.27			
Memory encoding (sum of 5 trials)	Senior dancers	53.00	8.03	0.32	.58	.01
	Control group	54.59	6.21			
Long-term memory (trial 5 – trial_delayed_)	Senior dancers	2.43	2.17	3.01	.09	.05
	Control group	1.45	1.57			

**Table 3:** M and SD for ICV-adjusted hippocampal volume of the left and right hemispheres separated for senior dancers (*n* = 28) and controls (*n* = 29), as well as *F*- and *p* values and effect sizes of the respective MANCOVA with age and IQ score as covariates.

Brain volume (in cm^3^)	Group	M	SD	*F*(1,55)	*p*	p*η* ^2^
Left hippocampus	Senior dancers	3.53	0.34	1.50	.23	.03
	Control group	3.64	0.33			
Right hippocampus	Senior dancers	3.81	0.38	0.54	.47	.01
	Control group	3.91	0.42			

**Table 4:** VBM8 results for contrast dancers > controls, revealing brain regions with larger volume in senior dancers than control participants.

Region	Cluster level		Peak level		MNI peak coordinates		
	*k*_*E*_	*p*_FWE-corr_	*Z*	*p*_uncorr_	*x*	*y*	*z*
Medial frontal G/BA 9	93	.818	3.85	.000	0	50	30
Middle frontal G/BA 10	72	.873	3.61	.000	−29	53	10
Superior frontal G/BA 9	36	.951	3.76	.000	29	50	30
Medial frontal G/BA 10	5	.992	3.36	.000	3	59	16
Superior frontal G/BA 8	25	.969	3.30	.000	3	35	45
Middle frontal G/BA 10	6	.991	3.21	.001	38	57	12
Medial frontal G/BA 10	2	.995	3.18	.001	−2	57	16
Medial frontal G/BA 10	10	.987	3.18	.001	−20	48	13

Note: G = gyrus, BA = Brodmann area, and *k*_*E*_ = cluster extent in voxel.

**Table 5:** Pearson product-moment correlation coefficient (*r*) and significance level (*p*) between individual cognitive performances in the four cognitive tasks and individual brain volumes of the left and right hippocampi.

Cognitive task	Left hippocampus		Right hippocampus	
	*r*	*p*	*r*	*p*
Flanker Task (*q*-score)	.01	.46	−.04	.38
Visual Search Task (*q*-score)	.07	.31	−.02	.46
Memory encoding	<−.01	.49	.04	.39
Long-term memory	−**.30**	**.01**	−**.38**	**<.01**

## References

[1] Bherer L., Erickson K. I., Liu-Ambrose T. (2013). Physical exercise and brain functions in older adults. *Journal of Aging Research*.

[2] Colcombe S., Kramer A. F. (2003). Fitness effects on the cognitive function of older adults: a meta-analytic study. *Psychological Science*.

[3] Hamer M., Chida Y. (2009). Physical activity and risk of neurodegenerative disease: a systematic review of prospective evidence. *Psychological Medicine*.

[4] Erickson K. I., Voss M. W., Prakash R. S. (2011). Exercise training increases size of hippocampus and improves memory. *Proceedings of the National Academy of Sciences of the United States of America*.

[5] Maass A., Düzel S., Goerke M. (2015). Vascular hippocampal plasticity after aerobic exercise in older adults. *Molecular Psychiatry*.

[6] Niemann C., Godde B., Voelcker-Rehage C. (2014). Not only cardiovascular, but also coordinative exercise increases hippocampal volume in older adults. *Frontiers in Aging Neuroscience*.

[7] Voelcker-Rehage C., Godde B., Staudinger U. M. (2010). Physical and motor fitness are both related to cognition in old age. *The European Journal of Neuroscience*.

[8] Voelcker-Rehage C., Godde B., Staudinger U. M. (2011). Cardiovascular and coordination training differentially improve cognitive performance and neural processing in older adults. *Frontiers in Human Neuroscience*.

[9] Niemann C., Godde B., Staudinger U. M., Voelcker-Rehage C. (2014). Exercise-induced changes in basal ganglia volume and cognition in older adults. *Neuroscience*.

[10] Erickson K. I., Leckie R. L., Weinstein A. M. (2014). Physical activity, fitness, and gray matter volume. *Neurobiology of Aging*.

[11] Voelcker-Rehage C., Niemann C. (2013). Structural and functional brain changes related to different types of physical activity across the life span. *Neuroscience and Biobehavioral Reviews*.

[12] US Department of Health and Human Services (1996). *Physical Activity and Health: A Report of the Surgeon General*.

[13] Fabel K., Wolf S. A., Ehninger D., Babu H., Leal-Galicia P., Kempermann G. (2009). Additive effects of physical exercise and environmental enrichment on adult hippocampal neurogenesis in mice. *Frontiers in Neuroscience*.

[14] Verghese J., Lipton R. B., Katz M. J. (2003). Leisure activities and the risk of dementia in the elderly. *The New England Journal of Medicine*.

[15] Verghese J. (2006). Cognitive and mobility profile of older social dancers. *Journal of the American Geriatrics Society*.

[16] Alpert P. T., Miller S. K., Wallmann H. (2009). The effect of modified jazz dance on balance, cognition, and mood in older adults. *Journal of the American Academy of Nurse Practitioners*.

[17] Kattenstroth J.-C., Kolankowska I., Kalisch T., Dinse H. R. (2010). Superior sensory, motor, and cognitive performance in elderly individuals with multi-year dancing activities. *Frontiers in Aging Neuroscience*.

[18] Kattenstroth J.-C., Kalisch T., Holt S., Tegenthoff M., Dinse H. R. (2013). Six months of dance intervention enhances postural, sensorimotor, and cognitive performance in elderly without affecting cardio-respiratory functions. *Frontiers in Aging Neuroscience*.

[19] Fjell A. M., Walhovd K. B. (2010). Structural brain changes in aging: courses, causes and cognitive consequences. *Reviews in the Neurosciences*.

[20] Raz N., Lindenberger U., Rodrigue K. M. (2005). Regional brain changes in aging healthy adults: general trends, individual differences and modifiers. *Cerebral Cortex*.

[21] Li S.-C., Lindenberger U., Hommel B., Aschersleben G., Prinz W., Baltes P. B. (2004). Transformations in the couplings among intellectual abilities and constituent cognitive processes across the life span. *Psychological Science*.

[22] Folstein M. F., Folstein S. E., McHugh P. R. (1975). ‘Mini-mental state’. A practical method for grading the cognitive state of patients for the clinician. *Journal of Psychiatric Research*.

[23] Colcombe S. J., Kramer A. F., Erickson K. I. (2004). Cardiovascular fitness, cortical plasticity, and aging. *Proceedings of the National Academy of Sciences of the United States of America*.

[24] Baecke J. A. H., Burema J., Frijters J. E. R. (1982). A short questionnaire for the measurement of habitual physical activity in epidemiological studies. *American Journal of Clinical Nutrition*.

[25] Wagner P., Singer R. (2003). Ein fragebogen zur erfassung der habituellen körperlichen aktivität verschiedener bevölkerungsgruppen. *Sportwissenschaft*.

[26] Ainsworth B. E., Haskell W. L., Whitt M. C. (2000). Compendium of physical activities: an update of activity codes and MET intensities. *Medicine and Science in Sports and Exercise*.

[27] Ainsworth B. E., Haskell W. L., Leon A. S. (1993). Compendium of physical activities: classification of energy costs of human physical activities. *Medicine and Science in Sports and Exercise*.

[28] Aartsen M. J., Smits C. H. M., van Tilburg T., Knipscheer K. C. P. M., Deeg D. J. H. (2002). Activity in older adults: cause or consequence of cognitive functioning? A longitudinal study on everyday activities and cognitive performance in older adults. *The Journals of Gerontology, Series B: Psychological Sciences and Social Sciences*.

[29] Voelcker-Rehage C., Wiertz O. (2003). *Die Lernfähigkeit sportmotorischer Fertigkeiten im Lichte der Entwicklungspsychologie der Lebensspanne*.

[30] Adrian M. J., Smith E. L., Serfass R. C. (1981). Flexibility in the aging adult. *Exercises and Aging: The Scientific Basic*.

[31] Rikli R. E., Jones C. J. (2001). *Senior Fitness Test Manual. Development and Validation of a Functional Fitness Test for Community-Residing Older Adults*.

[32] Ehrler W., Huth M., Martin P., Ettrich K. U., Lehr U., Roether D., Fischer-Cyrulies A. (2000). Körperliche leistungsfähigkeit 43- bis 45-jähriger und 62- bis 64-jähriger im Vergleich. *Aspekte der Entwicklung im Mittleren und Höheren Lebensalter*.

[33] Kiphard E. J., Schilling F. (1974). *Körperkoordinationstest für Kinder*.

[34] Ekdahl C., Jarnlo G. B., Andersson S. I. (1989). Standing balance in healthy subjects. Evaluation of a quantitative test battery on a force platform. *Scandinavian Journal of Rehabilitation Medicine*.

[35] Tiffin J., Asher E. J. (1948). The Purdue Pegboard: norms and studies of reliability and validity. *Journal of Applied Psychology*.

[36] Helmstaedter C., Lendt M., Lux S. (2001). *Verbaler Lern- und Merkfähigkeitstest*.

[37] Winneke A. H., Godde B., Reuter E.-M., Vieluf S., Voelcker-Rehage C. (2012). The association between physical activity and attentional control in younger and older middle-aged adults: an ERP study. *The Journal of Gerontopsychology and Geriatric Psychiatry*.

[38] Gualtieri C. T., Johnson L. G. (2008). A computerized test battery sensitive to mild and severe brain injury. *Medscape General Medicine*.

[39] Hommel B., Li K. Z. H., Li S.-C. (2004). Visual search across the life span. *Developmental Psychology*.

[40] Erickson K. I., Prakash R. S., Voss M. W. (2009). Aerobic fitness is associated with hippocampal volume in elderly humans. *Hippocampus*.

[41] Flöel A., Ruscheweyh R., Krüger K. (2010). Physical activity and memory functions: are neurotrophins and cerebral gray matter volume the missing link?. *NeuroImage*.

[42] Ruscheweyh R., Willemer C., Krüger K. (2011). Physical activity and memory functions: an interventional study. *Neurobiology of Aging*.

[43] Hayes S. M., Hayes J. P., Cadden M., Verfaellie M. (2013). A review of cardiorespiratory fitness-related neuroplasticity in the aging brain. *Frontiers in Aging Neuroscience*.

[44] Raz N., Rodrigue K. M., Kennedy K. M., Head D., Gunning-Dixon F., Acker J. D. (2003). Differential aging of the human striatum: longitudinal evidence. *American Journal of Neuroradiology*.

[45] Ritchie S. J., Booth T., Valdés Hernández M. d. C. (2015). Beyond a bigger brain: multivariable structural brain imaging and intelligence. *Intelligence*.

[46] Basten U., Hilger K., Fiebach C. J. (2015). Where smart brains are different: a quantitative meta-analysis of functional and structural brain imaging studies on intelligence. *Intelligence*.

[47] Shvartz E., Reibold R. C. (1990). Aerobic fitness norms for males and females aged 6 to 75 years: a review. *Aviation Space and Environmental Medicine*.

[48] Berryman N., Bherer L., Nadeau S. (2013). Executive functions, physical fitness and mobility in well-functioning older adults. *Experimental Gerontology*.

[49] Berryman N., Bherer L., Nadeau S. (2014). Multiple roads lead to Rome: combined high-intensity aerobic and strength training vs. gross motor activities leads to equivalent improvement in executive functions in a cohort of healthy older adults. *Age*.

[50] Weinstein A. M., Voss M. W., Prakash R. S. (2012). The association between aerobic fitness and executive function is mediated by prefrontal cortex volume. *Brain, Behavior, and Immunity*.

[51] Yonelinas A. P. (2013). The hippocampus supports high-resolution binding in the service of perception, working memory and long-term memory. *Behavioural Brain Research*.

[52] Bugg J. M., Head D. (2011). Exercise moderates age-related atrophy of the medial temporal lobe. *Neurobiology of Aging*.

